# Acidemia predicts mortality independently of lactate levels in patients after cardiac arrest

**DOI:** 10.1016/j.resplu.2026.101234

**Published:** 2026-01-16

**Authors:** Dragos A. Duse, Andreea I. Ganea, Patrick Horn, Matthias Ortkemper, Jafer Haschemi, Philipp Deffke, Christian Jung, Malte Kelm, Ralf Erkens

**Affiliations:** a Department of Cardiology, Pulmonology, and Vascular Medicine, Medical Faculty, Heinrich Heine University, Duesseldorf, Germany; b Department of Anesthesiology and Operative Intensive Care Medicine, Faculty of Medicine, University of Cologne, Cologne, Germany; c Cardiovascular Research Institute Düsseldorf (CARID), Germany; d Clinic for Cardiology/Electrophysiology, St. Agnes-Hospital Bocholt, Klinikum Westmünsterland, Germany

**Keywords:** Cardiac arrest, Lactate, pH, Mortality, Neurological outcome

## Abstract

**Aim:**

We examined whether post-cardiac arrest acidemia is associated with 30-day mortality and neurological outcomes among hospital survivors, independent of lactate and partial arterial carbon dioxide pressure (paCO_2_) levels, in patients after cardiopulmonary resuscitation (CPR).

**Methods:**

The predictive value of acidemia for in-hospital mortality was analyzed retrospectively in 742 non-traumatic cardiac arrest patients admitted to a German high-volume tertiary center using receiver operating characteristic (ROC) analysis. Patients were stratified using the ROC-derived (Youden-optimal) pH cut-off, and 30-day mortality was compared across strata. Cox regression assessed the association between severe acidemia (pH ≤ 7.2, binary) and mortality and examined its consistency across prespecified subgroups (age, sex, cardiac arrest type, lactate, and paCO_2_). Preliminary findings were externally validated in a database containing over 2000 patients from multiple intensive care units (eICU database).

**Results:**

Admission pH levels predicted in-hospital mortality (area-under-curve 0.75, *p* < 0.0001). The ROC-derived Youden-optimal threshold was pH 7.207; for clinical interpretability, this value was rounded to pH 7.2 and used as the cut-off for severe acidemia. Patients with severe acidemia exhibited substantially higher in-hospital and 30-day mortality. Among hospital survivors, no statistically significant association between admission pH and neurological outcome was observed. In Cox models, pH ≤ 7.20 remained significantly associated with mortality independent of lactate, paCO_2_, arrest type, age, and sex. In the validation eICU cohort, pH ≤ 7.2 accurately stratified cardiac-arrest patients with a higher mortality risk. This association persisted in Cox regression analyses of subgroups stratified by lactate and paCO_2_ levels (all *p* < 0.0001), as proxies for systemic hypoperfusion and ventilation.

**Conclusion:**

Post-cardiac-arrest acidemia is associated with higher mortality independently of lactate, ventilation, or CPR characteristics. Among hospital survivors, admission pH was not significantly associated with neurological outcome. These findings support pH as an early marker for mortality risk stratification after cardiac arrest, to be interpreted in the context of multimodal prognostication.

## Introduction

Cardiac arrest occurs unexpectedly and represents one of the leading causes of death worldwide.^1^ Despite improved medical therapies in Western countries over the years,^2^ survival rates are low and range 6–22% for out-of-hospital cardiac arrest (OHCA)^3^, ^4^ and ∼33% for in-hospital cardiac arrest (IHCA).^5^ Only a small proportion of survivors recover with a good neurological status.^3^

Prognostication of outcomes during and after CPR is difficult and currently imprecise. Numerous CPR characteristics influence hypoxia and hypoperfusion duration, directly affecting outcomes. While no single standardized marker of tissue hypoxia exists, arterial lactate reflects systemic hypoperfusion,^6^ and paCO_2_ primarily reflects ventilatory status.^7^ Both markers fluctuate after CPR and are associated with mortality and neurological outcomes.^8^, ^9^, ^10^, ^11^, ^12^ Arterial pH integrates underlying acid-base disturbances and reflects the body's compensatory mechanisms via respiratory or metabolic pathways. This rationale has led to the development of novel risk scores that incorporate one or more of these markers (lactate, paCO_2_, and pH) alongside CPR-related variables to enhance prognostic accuracy. That was the case for scoring systems, such as MIRACLE2,^13^ Cardiac Arrest Hospital Prognosis (CAHP),^14^ OHCA,^15^ and TTM,^16^ which included one or more of these biomarkers and showed high accuracy in discriminating OHCA patients at risk of unfavorable outcomes in external validation studies.^17^ In addition, in cardiogenic shock patients, whose metabolic profiles resemble those observed after cardiac arrest, pH enhanced lactate's predictive value, even when accounting for stratifiable shock classes.^18^

Despite the availability of CPR risk scores, clinicians may still benefit from simple, readily applicable tools for early risk assessment and stratification. Pre-hospital blood gas analysis are increasingly available worldwide. We therefore tested whether post-arrest acidemia, quantified by the first available arterial pH, was associated with higher mortality independent of lactate, systemic hypoperfusion, and ventilatory status, and whether it could stratify neurological outcomes among hospital survivors.

## Methods

### Study design and populations

The primary objective of this study was to determine whether post-resuscitation acidemia predicts mortality across strata of markers of systemic hypoperfusion and ventilatory status. The secondary objective was to evaluate the association between admission pH and neurological outcome conditional on survival, i.e., among hospital survivors after cardiac arrest. For this purpose, we analyzed data from 1086 non-traumatic cardiac arrest patients treated at a high-volume tertiary center in Germany (University Clinic Duesseldorf) between January 1, 2013, and December 31, 2017. A detailed description of the database has been previously published.^19^ All patients were identified retrospectively based on documented CPR for non-traumatic cardiac arrest. Eligible patients were >18 years and had (i) a recorded episode of CPR with return of spontaneous circulation (ROSC) followed by admission to the intensive care unit (ICU) for post–cardiac arrest care, or (ii) CPR for non-traumatic cardiac arrest with death during initial treatment in the emergency department or catheterization laboratory. Case identification was performed using ICU admission records and resuscitation documentation. Patients with traumatic cardiac arrest or without an arterial blood gas obtained at admission were excluded. Because CPR, ROSC status, and subsequent post–cardiac arrest management are explicitly documented in our institution, the risk of misclassifying non–cardiac arrest patients as cardiac arrest cases is considered very low. All patients from the database were followed up to either in-hospital death or hospital discharge. Post-CPR lactate and paCO_2_ levels were utilized as surrogate markers for systemic hypoperfusion and impaired ventilation. Differentiation between impaired and compensatory ventilation was not possible based on the available data. We restricted our analyses to patients with documented values for pH, lactate, and available clinical outcomes.

Data collection was approved by the ethics committee of Heinrich-Heine University (2018-109-RetroDEuA). Due to the study's retrospective nature, informed consent from patients was not required. This study was conducted in accordance with the Declaration of Helsinki.

Preliminary findings were validated in an independent cohort from the eICU Collaborative Research Database, a large, multicenter dataset containing information on over 8500 patients from more than 300 intensive care units in the United States. Detailed information on the database has been previously provided elsewhere.^19^, ^20^ In the analysis of the eICU collective, only patients with available lactate and pH values from the first intrahospital day following CPR were included.

### Data collection

Arterial pH, lactate, and paCO_2_ were derived from the first available arterial blood gas sample after cardiac arrest. For OHCA, sampling occurred immediately on hospital arrival and could be performed during ongoing CPR (pre-ROSC) or immediately after ROSC; for IHCA, arterial blood gas analyses were typically obtained during ongoing in-hospital CPR and, when applicable, immediately after ROSC during initial post-resuscitation stabilization. Values from the initial blood gas analysis, hospitalization days following CPR, and neurological status at discharge were collected from medical records in the hospital's clinical data information systems (Medico (Cerner GmBh Deutschland) and PEGASOS (Nexus/Marabu GmBh)). Neurological status was assessed using the cerebral performance category (CPC). The detailed CPC scale has been previously described elsewhere.^21^ A favorable neurological status was assumed for CPC scores 1–2, while a CPC score of 3–4 was assumed for an unfavorable neurological status. Given the high overall mortality after cardiac arrest, analyses of neurological outcomes were restricted to hospital survivors (patients with CPC scores 1–4) to focus on neurological function among survivors and avoid an endpoint dominated by CPC 5 (death). No a priori power calculation was performed for this secondary endpoint.

Specific baseline characteristics, demographic data, and various laboratory parameters, including initial serum lactate and paCO_2_ levels on the first day post-CPR, were collected from the multicenter eICU collective and analyzed accordingly. We used the maximum lactate and paCO_2_ values and the minimum pH value for patients with multiple documented values on the same day.

### Statistical analysis

The presented results are displayed as mean ± standard error of the mean (SEM). Unless otherwise specified, we used an unpaired Student's *t*-test (for normally distributed data) or a Mann-Whitney *U* test (for non-normally distributed data) to test whether the two groups of continuous data differed significantly. Normality was tested using the D'Agostino-Pearson test. Similarly, statistical differences between groups of categorical data were assessed by the Chi-square test. The prognostic value of lactate and pH levels was analyzed using ROC curves, and the accuracy was assessed by the area under the curve (AUC). ROC-derived Youden-optimal cut-offs were calculated for pH and lactate; the pH cut-off was rounded to 7.2 for interpretability and used for subsequent stratified analyses. Survival differences were illustrated using Kaplan-Meier curves, and statistical significance was assessed using the log-rank test. The association between admission pH and mortality was assessed using Cox proportional hazards regression with pH modeled as a binary variable (pH ≤ 7.2 vs >7.2), and consistency was evaluated across prespecified strata defined by age, sex, arrest setting (IHCA vs OHCA), lactate, and paCO_2_. The strength of associations was expressed as hazard ratios (HRs) with corresponding 95% confidence intervals. In addition, to examine whether pH provided prognostic information beyond lactate and paCO_2_, we fitted a mutually adjusted Cox model including pH, lactate, and paCO_2_ simultaneously. A two-tailed *p*-value <0.05 was considered statistically significant. All statistical analyses were conducted using GraphPad Prism 9.3.0 for Windows (GraphPad Software, San Diego, CA, USA).

## Results

### Patients

**Fig. 1:**
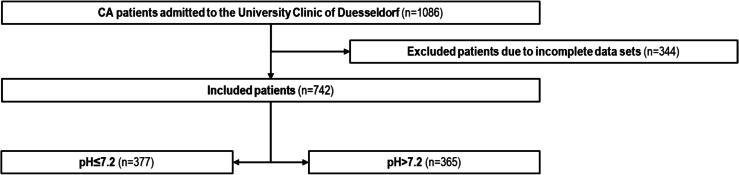
**Study flow chart**. Of 1086 cardiac arrest patients admitted to the University Hospital Düsseldorf, 344 were excluded due to incomplete datasets, leaving 742 patients for analysis. Patients were stratified by admission pH into pH ≤ 7.2 (*n* = 377) and pH > 7.2 (*n* = 365).

Of the 1086 patients initially screened, 344 had no documented initial lactate levels or pH values and were excluded from the analysis. The remaining 742 patients were included in the study (Fig. 1). The mean age was 70 years, and nearly two-thirds of the patients in the group were male (*n* = 465, 63%). In over 75% of cases, the arrest was witnessed. A shockable heart rhythm was encountered in one-quarter of the cases (*n* = 194). The renal function at admission was often impaired. The mean lactate was 9.6 mmol/l, and the mean pH 7.2. Despite a high rate of ROSC (78%), in-hospital mortality was high at 74%. Among survivors, ∼20% were discharged with a favorable neurological outcome (CPC score 1–2). The complete baseline characteristics are shown in SI Table 1.

### Acidemia is associated with mortality, while no statistically significant association with neurological outcome was observed among hospital survivors

**Fig. 2:**
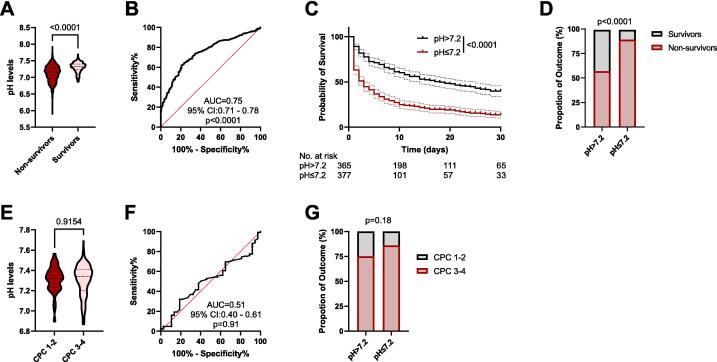
**Admission pH and outcomes after cardiac arrest**. (A) Admission pH in survivors versus non-survivors at hospital discharge (*n*_Suvivors_ = 196, *n*_Non-suvivors_ = 546). Mann–Whitney *U* test, *p*-value shown. (B) ROC curve of admission pH for in-hospital mortality (AUC: 0.75, 95%-CI: 0.71–0.78, *p* < 0.0001). Youden Index: 7.207. (C) Kaplan–Meier analysis of 30-day survival stratified by admission pH (≤7.2 vs >7.2); log-rank (Mantel–Cox) test, *p*-value shown. (D) Proportion of survivors and non-survivors by pH category; (*n*_pH>7.2_ = 365, *n*_pH>7.2_ = 377). χ^2^ test, *p*-value shown. (E) Among hospital survivors with available CPC data, admission pH by favorable (CPC 1–2) versus unfavorable (CPC 3–4) neurological outcome (*n*_CPC 1–2_ = 37, *n*_CPC 3–4_ = 125); Mann–Whitney *U* test, *p*-value shown. (F) ROC curve of admission pH for favorable neurological outcome among survivors (AUC 0.51, 95% CI 0.40–0.61; *p* = 0.91). (G) Proportion of favorable versus unfavorable neurological outcome among survivors stratified by admission pH (≤7.2 vs >7.2); (*n*_pH>7.2_ = 126, *n*_pH>7.2_ = 36). χ^2^ test, *p*-value shown.

Among 742 patients, initial pH levels were significantly lower in non-survivors than in survivors (*p* < 0.0001; Fig. 2A). The predictive value of pH for in-hospital mortality was confirmed by an area under the receiver operating characteristic curve (AUROC) of 0.75 (95% CI: 0.71–0.78, *p* < 0.0001; Fig. 2B). The ROC-derived optimal pH cut-off (Youden index) was 7.207; for subsequent stratified analyses, patients were grouped using pH ≤ 7.2. The Kaplan–Meier survival analysis of the first 30-day and the analysis of in-hospital mortality showed significantly reduced survival in patients with pH ≤ 7.2 (*p* < 0.0001; Fig. 2C and D).

Among hospital survivors, pH levels did not differ statistically significant between those with favorable (CPC 1–2) and unfavorable (CPC 3–4) neurological outcomes (*p* = 0.62; Fig. 2E). Similarly, pH showed no discriminative power for neurological outcome in ROC analysis (AUC = 0.51, 95% CI: 0.40–0.61, *p* = 0.91; Fig. 2F), and no significant proportion of favorable or unfavorable outcomes were observed across the stratification by the pH cut-off of 7.2 (14% in pH ≤ 7.2 versus 25% in pH > 7.2, *p* = 0.18; Fig. 2G).

### Robust association between acidemia and mortality independent of systemic hypoperfusion or ventilation

**Fig. 3:**
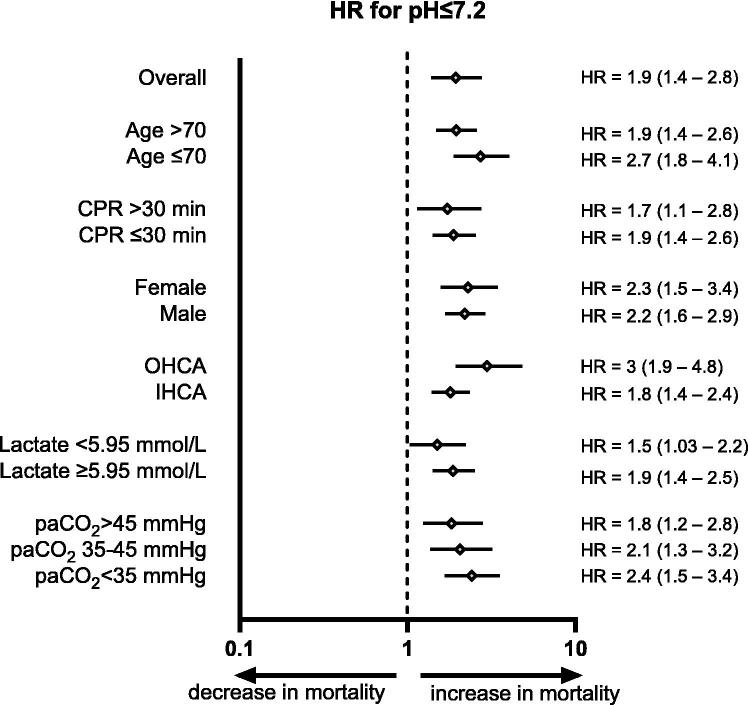
**pH ≤ 7.2 is associated with a higher mortality**. Forest plot showing hazard ratios (HRs) for mortality in patients with pH ≤ 7.2 across the entire collective and predefined clinical subgroups. Diamonds represent the point estimate of the HR, and horizontal lines indicate the 95% confidence intervals. Subgroups include age (>70 vs. ≤70 years), duration of CPR (>30 min vs. ≤30 min), sex, cardiac arrest location (out-of-hospital cardiac arrest [OHCA] vs. in-hospital cardiac arrest [IHCA]), lactate levels (<5.95 vs. ≥5.95 mmol/L), and arterial carbon dioxide tension (paCO_2_ > 45, 35–45, or <35 mmHg). HRs > 1 suggest increased mortality risk associated with severe acidemia (pH ≤ 7.2), while HRs < 1 indicate a potential survival benefit.

In stratified Cox regression analyses, pH ≤ 7.2 was associated with increased mortality in the entire cohort and clinically relevant subgroups, including age, CPR duration, sex, and arrest characteristics (Fig. 3).

Lactate discriminated mortality risk with an AUROC of 0.83 (95% CI: 0.79–0.86, *p* < 0.0001). The ROC-derived optimal cut-off (Youden index) was 5.95 mmol/L. In Cox regression analysis, pH ≤ 7.2 was associated with increased mortality risk for both lactate <5.95 mmol/L (HR = 1.5, 95% CI: 1.03–2.2) and ≥5.95 mmol/L (HR = 1.9, 95% CI: 1.4–2.5) groups. Combined stratification by pH (≤7.2) and lactate (≥5.95 mmol/L) further separated mortality risk (Kaplan–Meier analysis; Supplementary Fig. S1A). The lowest survival was observed in patients with both acidemia and elevated lactate, whereas the highest survival was observed in patients with pH > 7.2 and lactate < 5.95 mmol/L (log-rank *p* < 0.0001).

The association between acidemia (pH ≤ 7.2) and mortality was also assessed across paCO_2_ strata. Patients were categorized as hypocapnic (paCO_2_ < 35 mmHg), normocapnic (paCO_2_ between 35 and 45 mmHg), and hypercapnic (paCO_2_ > 45 mmHg). pH ≤ 7.2 was associated with higher mortality in each paCO_2_ category (Fig. 3, hypocapnia: HR = 2.4, 95% CI: 1.5–3.4; normocapnia: HR = 2.1, 95% CI: 1.3–3.2, and hypercapnia: HR = 1.8, 95% CI: 1.2–2.8). In the multivariable Cox model including pH, lactate, and paCO_2_, lower pH and higher lactate were independently associated with increased mortality (pH per unit: HR 0.23, 95% CI 0.14–0.36; lactate per unit: HR 1.08, 95% CI 1.06–1.10; both *p* < 0.0001, Table 1), whereas paCO_2_ showed no statistically significant association (per unit: HR 0.99, 95% CI 0.99–1.00; *p* = 0.0566, Table 1).

**Table 1:** **Multivariable Cox proportional hazards model for mortality including admission pH, lactate, and paCO_2_.** Hazard ratios (HRs) with 95% confidence intervals and *p*-values are shown for each covariate entered simultaneously in the model (Efron method for ties). Continuous predictors are expressed per 1-unit increase in the respective variable (pH units, mmol/L for lactate, and mmHg for paCO_2_); HR < 1 indicates lower hazard with increasing values.

**Variable**	**Hazard ratios**	**95% CI**	***p*-value**
pH	0.23	0.14–0.36	<0.0001
Lactate	1.08	1.06–1.10	<0.0001
paCO_2_	0.99	0.99–1.00	0.0566

### Validation of the predictive role of pH additive to lactate

**Fig. 4:**
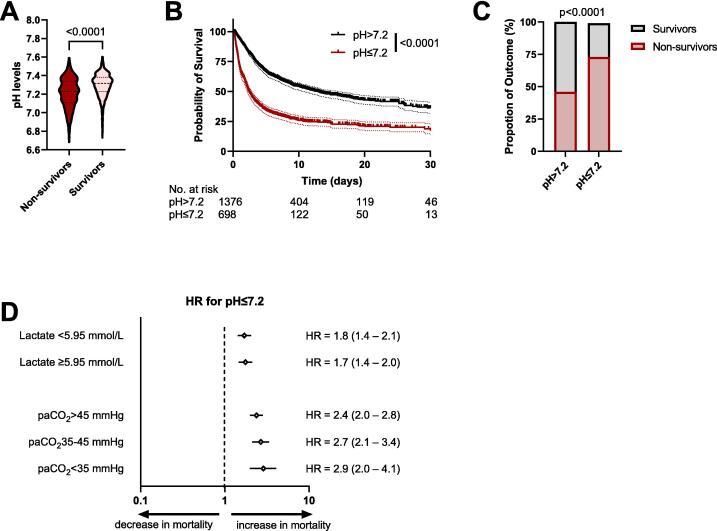
**External validation confirms acidemia as an independent predictor of mortality in the eICU cohort**. (A) Comparison of pH levels between cardiac arrest survivors and non-survivors from the eICU cohort. *n*_Suvivors_ = 931, *n*_Non-suvivors_ = 1143. Mann–Whitney *U* test, *p*-value shown. (B) Kaplan-Meier survival analysis of the 30-day mortality of cardiac arrest patients from the eICU database stratified by pH; log-rank (Mantel–Cox) test, *p*-value shown. (C) The proportion of survivors and non-survivors in each pH stratification group within the eICU database. χ^2^ test, *p*-value shown. (D) Forest plot showing hazard ratios (HRs) for mortality in patients with pH ≤ 7.2 across predefined clinical subgroups in patients from the eICU database. The reference line at HR = 1 indicates no difference in risk. Diamonds represent the point estimate of the HR, and horizontal lines indicate the 95% confidence intervals. Subgroups include lactate levels (<5.95 vs. ≥5.95 mmol/L), and arterial carbon dioxide tension (paCO_2_ > 45, 35–45, or <35 mmHg).

To assess external validity, we analyzed 2074 post–cardiac arrest patients from the eICU Collaborative Research Database. Baseline characteristics are summarized in SI Table 2. Non-survivors had lower initial pH values than survivors (*p* < 0.0001; Fig. 4A). In Kaplan–Meier analysis, pH ≤ 7.2 was associated with reduced 30-day survival (log-rank *p* < 0.0001; Fig. 4B). Consistently, pH ≤ 7.2 was associated with lower in-hospital survival (*p* < 0.0001; Fig. 4C).

In the eICU cohort, pH ≤ 7.2 was associated with mortality within lactate strata (lactate <5.95 mmol/L: HR 1.8, 95% CI 1.4–2.1, *p* < 0.0001; lactate ≥5.95 mmol/L: HR 1.7, 95% CI 1.4–2.0, *p* < 0.0001). Combined stratification by pH and lactate further separated risk groups (Supplementary Fig. S1B). Similar associations were observed across PaCO_2_ strata, including hypocapnia (<35 mmHg: HR 2.9, 95% CI: 2.0–4.1), normocapnia (35–45 mmHg: HR 2.7, 95% CI: 2.1–3.4), hypercapnia (>45 mmHg: HR 2.4, 95% CI: 2.0–2.8), and (Fig. 4D).

## Discussion

In this study, admission arterial pH was associated with increased mortality after cardiac arrest. The association persisted when accounting for markers of systemic hypoperfusion (lactate) and ventilatory status (paCO_2_) and was consistent across clinically relevant strata and prespecified subgroups (including age, sex, arrest setting, and CPR duration). Using the clinically established threshold for severe acidemia (pH ≤ 7.20), pH-based stratification provided an interpretable separation of mortality risk, and the association was reproduced in an independent external cohort from the eICU Collaborative Research Database. These findings support admission pH as a simple early marker for mortality risk stratification after cardiac arrest; however, pH should not be used in isolation to guide early treatment limitation decisions and should be interpreted within a multimodal prognostication framework. Among hospital survivors, admission pH was not significantly associated with neurological outcome; this analysis was limited by reduced sample size and survivor bias and should therefore be interpreted cautiously. Prospective studies should assess whether earlier, prehospital pH measurement can meaningfully improve risk stratification without promoting premature prognostic decisions.

Association between pH and lactate with mortality is not new. Lactate levels have demonstrated consistent accuracy as predictors of outcomes in most critical care conditions, including ST-elevation myocardial infarction, sepsis, several types of shock, and cardiac arrest.^8^, ^10^, ^22^, ^23^, ^24^, ^25^ Several studies reported some predictive lactate value in cardiogenic shock after 8 or 24 h.^26^, ^27^ In addition, not only one-time measurements but also the lactate clearance can be used in outcome prediction,^28^, ^29^ since its dynamics reflect acute metabolic alterations during sustained shock or after ROSC. At the same time, studies have attributed a prognostic significance to pH in the context of cardiac arrest. Notably, in a multicenter retrospective analysis of over 2200 OHCA patients, pH levels from initial blood gas analyses were associated with in-hospital survival.^30^ A retrospective study on 79 OHCA patients showed that venous blood pH following ROSC was associated with in-hospital mortality.^31^ The authors concluded that an independent association with a higher mortality risk in patients with a pH < 7.2^31^ is consistent with the threshold used in our study. However, venous blood undergoes acid-basic changes evoked by the peripheric vasculature and tissue and does not accurately reflect the metabolic state; therefore, any interpretation warrants caution. In a further comparison of cardiac arrest patients with and without ROSC, significant differences were observed in pH, lactate, and paCO_2_ levels. Among these, only paCO_2_ remained independently associated with sustained ROSC in multivariable logistic regression analyses.^32^

Acidosis initially results from respiratory disturbances and impaired gas exchange. In the following stages, the acid-base equilibrium cannot sustain the needed buffering mechanism, and acidosis worsens. Interestingly, acidosis itself can modulate cardiac hemodynamics. In experimental studies on dogs, infusing lactic acid depressed left-ventricular circulation, while right-ventricular contractility and pulmonary pressure rose.^33^ Hearts of guinea pigs responded less to levosimendan and adrenaline in acidotic milieus.^34^ Despite the lack of clinical studies to confirm these experimental findings, translational approaches on isolated trabeculae from end-stage heart failure patients showed loss of contractility and depressed β-adrenergic force response due to acidemia.^35^ Pieces of evidence point out a link between lactic acidosis and cardiomyocyte dysfunction, presented in detail elsewhere.^36^ Collectively, there is an established association between pH and lactate, with elevated levels adversely affecting cardiac hemodynamics. Plenty of data underlines our supposition that impaired systemic hypoperfusion and ventilation are linked to the mortality risk, and considering pH levels as the pilar between metabolic acidosis and compensatory hyperventilation might augment post-arrest prognostication.

pH, paCO_2_, and lactate are included among the predictor variables used in the prognostication scores MIRACLE2, CAHP, and TTM.^13^, ^14^, ^16^ Interestingly, these scores were primarily developed to predict neurological outcomes. All scores received external validation by showing a good prediction of death or poor neurological outcomes, with AUC ranging from 0.77 to 0.835,^17^ exceeding the AUROC of pH alone in our study. In addition, prior studies have reported associations between admission pH and neurological outcomes after cardiac arrest. In a three-center OHCA cohort, admission pH predicted a neurological endpoint in multivariable analysis; however, the endpoint included CPC 5 (death), thereby capturing a composite of survival and neurological status rather than neurological function among survivors.^30^ In cohorts with very high in-hospital mortality, such composite CPC-based endpoints may therefore be driven predominantly by the mortality component, making it challenging to isolate the relationship between pH and neurological disability conditional on survival. Similarly, a Japanese study of 372 OHCA patients found higher admission pH among patients with favorable neurological outcome at discharge, but overall survival was only 12%, again limiting the ability to separate mortality from neurological prognosis.^37^ Against this background, our data add a complementary perspective by examining neurological status conditional on survival (hospital survivors), thereby separating survival from functional neurological outcome in this high-mortality cohort; this survivor-only analysis, however, remains exploratory and is subject to limited power and survivor bias. Interestingly, paCO_2_ has been consistently associated with neurological outcomes, with both hypo- and hypercapnia linked to worse prognoses.^12^, ^38^ Our data might lay the groundwork for future analysis by incorporating pH, lactate, and paCO_2_ in a mutually informative manner to identify patients with the best neurological outcomes.

While validation by the second database enhanced the scientific quality of our findings, the strict retrospective design and the associated lack of causal justification must be considered significant limitations. In addition, we dichotomized pH using the ROC-derived optimal cut-off, as a clinically actionable threshold is more readily applicable in practice; however, this approach reduces granularity and may obscure non-linear associations, and future studies should therefore model pH continuously (e.g., using restricted cubic splines). Neurological outcome analyses were restricted to hospital survivors to evaluate functional neurological status conditional on survival in this high-mortality cohort. This resulted in a substantially smaller effective sample size and increased the risk of type II error. Moreover, restricting analyses to survivors introduces unavoidable survivor (selection) bias, as patients with more severe acidemia are more likely not to survive to discharge and therefore are not represented in survivor-only neurological comparisons. Thus, the survivor-only analysis cannot determine whether admission pH is associated with neurological injury independent of its association with mortality. Accordingly, the neurological outcome findings should be interpreted cautiously and cannot be generalized to the entire post–cardiac arrest population; no a priori power calculation was performed for this secondary endpoint. Because CPC is a coarse clinical scale, misclassification at category borders and ceiling effects (limited sensitivity to subtle cognitive deficits) may have attenuated observable associations. Lastly, sampling of blood gas analyses was heterogeneous with respect to timing (during ongoing CPR vs. immediately after ROSC), particularly in OHCA patients sampled on hospital arrival, whereas IHCA samples were typically obtained during in-hospital CPR. In addition, physiological and procedural differences between OHCA and IHCA may contribute to variability in pH, lactate, and paCO_2_, and arrest setting was not fully accounted for in all analyses. Future studies should assess the validity of our data prospectively, in addition to the question remaining open of whether balancing acidosis could improve prognosis.

## Conclusions

In a large single-center post–cardiac arrest cohort and an independent external validation cohort from the eICU Collaborative Research Database, admission acidemia (pH ≤ 7.2) was consistently associated with increased short-term mortality. This association remained evident across clinically relevant subgroups and strata reflecting systemic hypoperfusion (lactate) and ventilation (paCO_2_), and combined stratification by pH and lactate further separated risk categories. Among hospital survivors, no statistically significant association between admission pH and neurological outcome was observed; however, this analysis was limited by the small survivor sample size and unavoidable survivor bias, and should therefore be interpreted with caution and not generalized to the overall post–cardiac arrest population. These findings support pH ≤ 7.2 as an early, readily available marker for mortality risk stratification after cardiac arrest, suggesting increased in-hospital mortality.

## Data statement

The datasets used and analyzed during the current study are available from the corresponding author upon reasonable request.

## Declaration of AI and AI-assisted technologies in the writing process

During the preparation of this work, the authors used AI-assisted language tools (Grammarly, ChatGPT) in order to edit for clarity and coherence. After using this tool/service, the authors reviewed and edited the content as needed and take full responsibility for the content of the publication.

## CRediT authorship contribution statement

**Dragos A. Duse:** Writing – original draft, Visualization, Investigation, Formal analysis, Data curation, Conceptualization. **Andreea I. Ganea:** Investigation, Data curation, Conceptualization. **Patrick Horn:** Writing – review & editing, Validation. **Matthias Ortkemper:** Investigation. **Jafer Haschemi:** Investigation. **Philipp Deffke:** Investigation. **Christian Jung:** Writing – review & editing, Supervision, Investigation. **Malte Kelm:** Writing – review & editing, Validation, Supervision. **Ralf Erkens:** Writing – review & editing, Validation, Supervision, Methodology, Conceptualization.

## Funding

This research did not receive any specific grant from funding agencies in the public, commercial, or not-for-profit sectors.

## Declaration of competing interest

The authors declare that they have no known competing financial interests or personal relationships that could have appeared to influence the work reported in this paper.
