# Supplementary material for: Acidemia predicts mortality independently of lactate levels in patients after cardiac arrest
Source: Resusc Plus. 2026 Jan 16;28:101234. doi: 10.1016/j.resplu.2026.101234 (PMC12870871; doi:10.1016/j.resplu.2026.101234)
Supplement: Supplementary Data 1 [file mmc1.docx]

*Supplementary Information*

**Acidemia Predicts Mortality Independently of Lactate Levels in Patients After Cardiac Arrest**

Dragos A. Duse ^a^, Andreea I. Ganea ^a,b^, Patrick Horn ^a^, Matthias Ortkemper ^a^, Jafer Haschemi ^a^, Philipp Deffke ^a^, Christian Jung ^a,c^, Malte Kelm ^a,c^, Ralf Erkens ^a, d^

^a^ Department of Cardiology, Pulmonology, and Vascular Medicine, Medical Faculty, Heinrich Heine University, Duesseldorf, Germany.

^b^ Department of Anesthesiology and Operative Intensive Care Medicine, Faculty of Medicine, University of Cologne, Cologne, Germany.

^c^ Cardiovascular Research Institute Düsseldorf (CARID).

^d^ Clinic for Cardiology/Electrophysiology, St. Agnes-Hospital Bocholt, Klinikum Westmünsterland, Germany.

Corresponding author:

Dragos A. Duse, MD

Department of Cardiology, Pulmonology, and Vascular Medicine

University Hospital Düsseldorf

Heinrich Heine University, Düsseldorf, Germany

E-mail: dragos-andrei.duse@med.uni-duesseldorf.de

| **Characteristic** | **UKD collective** |
| --- | --- |
| n | 742 |
| Patient characteristics | |
| Age (years), mean | 70 |
| Sex (male), n (%) | 465 (63) |
| CA characteristics, n (%) | |
| Witnessed arrest | 577 (78) |
| Cardiac etiology of arrest | 277 (37) |
| Bystander CPR | 553 (75) |
| Shockable rhythm at the scene | 194 (26) |
| Target temperature management | 127 (17) |
| Outcomes | |
| Return of spontaneous circulation | 581 (78) |
| In-hospital mortality | 546 (74) |
| Favorable neurological outcome (CPC 1–2)* | 38 (19) |
| Laboratory parameters, mean±SD | |
| Sodium [mmol/l] | 142±9 |
| Potassium [mmol/l] | 4.9±2.6 |
| Creatinine [mg/dl] | 1.9±1.7 |
| eGFR [ml/min] | 48±27 |
| Hemoglobin [mmol/L] | 11±3 |
| INR | 2.1±3 |
| Lactate [mmol/L] | 9.6±6.4 |
| pH | 7.2±0.2 |
| paCO_2_ [mmHg] | 50±24 |
| paO_2_ [mmHg] | 180±142 |
| Base Excess [mmHg] | -9±10 |
| **analysis performed only among survivors* | |

**SI Table 1:** Baseline characteristics of the UKD collective.

| **Characteristics** | **eICU collective** |
| --- | --- |
| n | 2074 |
| Patient characteristics | |
| Age (years), mean | 64 |
| Sex (male), n (%) | 1188 (57) |
| Ethnicity, n (%) | |
| African American | 308 (15) |
| Asian | 29 (1) |
| Caucasian | 1552 (75) |
| Hispanic | 45 (2) |
| Native American | 18 (1) |
| Other/Unknown | 122 (6) |
| Laboratory parameters, mean±SD | |
| Sodium [mmol/l] | 138±5.6 |
| Potassium [mmol/l] | 3.7±0.8 |
| Creatinine [mg/dl] | 1.9±1.7 |
| Hemoglobin [mmol/L] | 11±2.7 |
| Lactate [mmol/L] | 5.6±5.1 |
| pH | 7.25±0.15 |
| paCO_2_ [mmHg] | 47±16 |
| paO_2_ [mmHg] | 103±71 |

**SI Table 2:** Baseline characteristics of the eICU collective.

**SI Figure 1: Combined stratification by pH and lactate levels improves mortality risk discrimination.** Kaplan–Meier survival curves showing 30-day mortality stratified by both arterial pH (≤7.2 vs. >7.2) and lactate (lac) levels (<5.95 mmol/L vs. ≥5.95 mmol/L), all *p*<0.0001, Log-rank test). (A) Survival analysis in the primary cohort (University Hospital Düsseldorf) revealed distinct risk profiles based on combined pH and lactate thresholds (B) Validation of the findings in the external eICU cohort confirmed consistent survival stratification (p < 0.0001, Log-rank test). Patients with both acidemia and elevated lactate (pH ≤ 7.2 and lactate ≥ 5.95 mmol/L; red line) had the lowest survival, while those with normal pH and lactate (green line) had the most favorable outcome. Intermediate groups (blue and orange lines) exhibited stepwise separation of survival curves. Number-at-risk tables are shown below each graph.
